# Rifampin-Induced Acute Intravascular Hemolysis Leading to Heme Pigment-Related Kidney Injury

**DOI:** 10.7759/cureus.9120

**Published:** 2020-07-10

**Authors:** Chandra Sanwal, Amber Kaldas, Salim Surani, Michael Bailey

**Affiliations:** 1 Internal Medicine, Corpus Christi Medical Center, Corpus Christi, USA; 2 Internal Medicine, University of North Texas, Dallas, USA; 3 Pathology, Corpus Christi Medical Center, Corpus Christi, USA

**Keywords:** acute tubular necrosis, acute interstitial nephritis, immune hemolytic anemia, rifampin, hemolysis, drug-induced acute renal failure

## Abstract

Rifampin-induced acute kidney injury is very rare. Most cases of acute renal injury from rifampin use are related to acute tubular necrosis and acute interstitial nephritis. In this case report, we detail a unique presentation of rifampin-associated acute intravascular hemolysis and subsequent tubular injury in a tuberculosis patient. The patient had presented to the hospital with acute kidney injury and oliguria from intravascular volume depletion secondary to intractable vomiting. The patient had stopped taking his antituberculosis medications two weeks before hospitalization. At the time of hospital admission, his antituberculosis regimen of rifampin and isoniazid was reinstituted. Within four days of initiation of rifampin, he developed acute hemolytic anemia. His kidney biopsy revealed hemoglobin pigment deposition in the kidney tubules. Rifampin was discontinued, and he received a total of eight hemodialysis treatments spanning over 17 days. Subsequently, after discontinuing rifampin, his anemia and oliguria resolved with renal function markedly improved to near normal baseline levels. This case report also offers a review of known mechanisms of rifampin-induced acute hemolysis and acute renal failure, along with a discussion of contemporary literature.

## Introduction

Drugs were first suspected as a cause of immune-hemolytic anemia (IHA) in 1953 [1]. Drug-induced thrombocytopenia is more common than drug-induced immune-hemolytic anemia (DIIHA). There is good data for the incidence of drug-induced thrombocytopenia (10-18 cases per million) and neutropenia (2-15 cases per million) but only crude estimates for DIIHA of about one per million of the population. Compared to DIIHA, autoimmune hemolytic anemia (AIHA) prevalence is higher and is reportedly one per hundred thousand of the population. The number of drugs causing DIIHA has increased from 15 known drugs in 1967 to a modest number of 125 in 2010. DIIHA is caused by IgM or IgG antibodies and seems to have no relationship to other allergic reactions (e.g., anaphylaxis), which are usually associated with IgE antibodies [1]. In a study by Mayer et al., among 73 patients with drug-induced hemolytic anemia, the most common single drugs identified were diclofenac, piperacillin, ceftriaxone, and oxaliplatin [2].

Rifampin is widely used in multidrug regimens for the treatment of tuberculosis (TB) and nontuberculous mycobacterial infections, and is also considered an effective antistaphylococcal agent. Hepatitis, thrombocytopenia, cutaneous syndrome, flu syndrome, abdominal syndrome, respiratory syndrome, orange-colored urine, and even disseminated intravascular coagulation (DIC) are some of the known side effects of rifampin [3-5]. Rifampin-associated acute renal failure (RARF) is a complication of anti-TB treatment occurring in less than 0.1% of patients with TB [6].

Rifampin treatment regimens are classified as ‘‘continuous,’’ with a daily intake of a rifampin dose; ‘‘intermittent,’’ with ingestion of a dose one, two, three, or five times weekly; and ‘‘interrupted,’’ when therapy is resumed after a course of daily or intermittent treatment and a subsequent drug-free interval [7]. In any case, it is widely accepted that the vast majority of RARF events are due to intermittent or interrupted rifampin use, for instance, in patients with previous drug exposure or poor compliers [6-8]. However, cases of RARF after continuous use of rifampin have been also reported [6,7]. There are multiple mechanisms of RARF: acute tubular necrosis (ATN), acute interstitial nephritis (AIN), rapidly progressive glomerulonephritis (RPGN), and light chain proteinuria. ATN from rifampin use can happen from heme pigment deposition due to intravascular hemolysis alone or from direct renal tubular damage [7,9]. Our case report is a rare presentation of RARF due to rifampin use in the interrupted manner, due to heme pigment deposition in renal tubules from intravascular hemolysis.

## Case presentation

**Figure 1:**
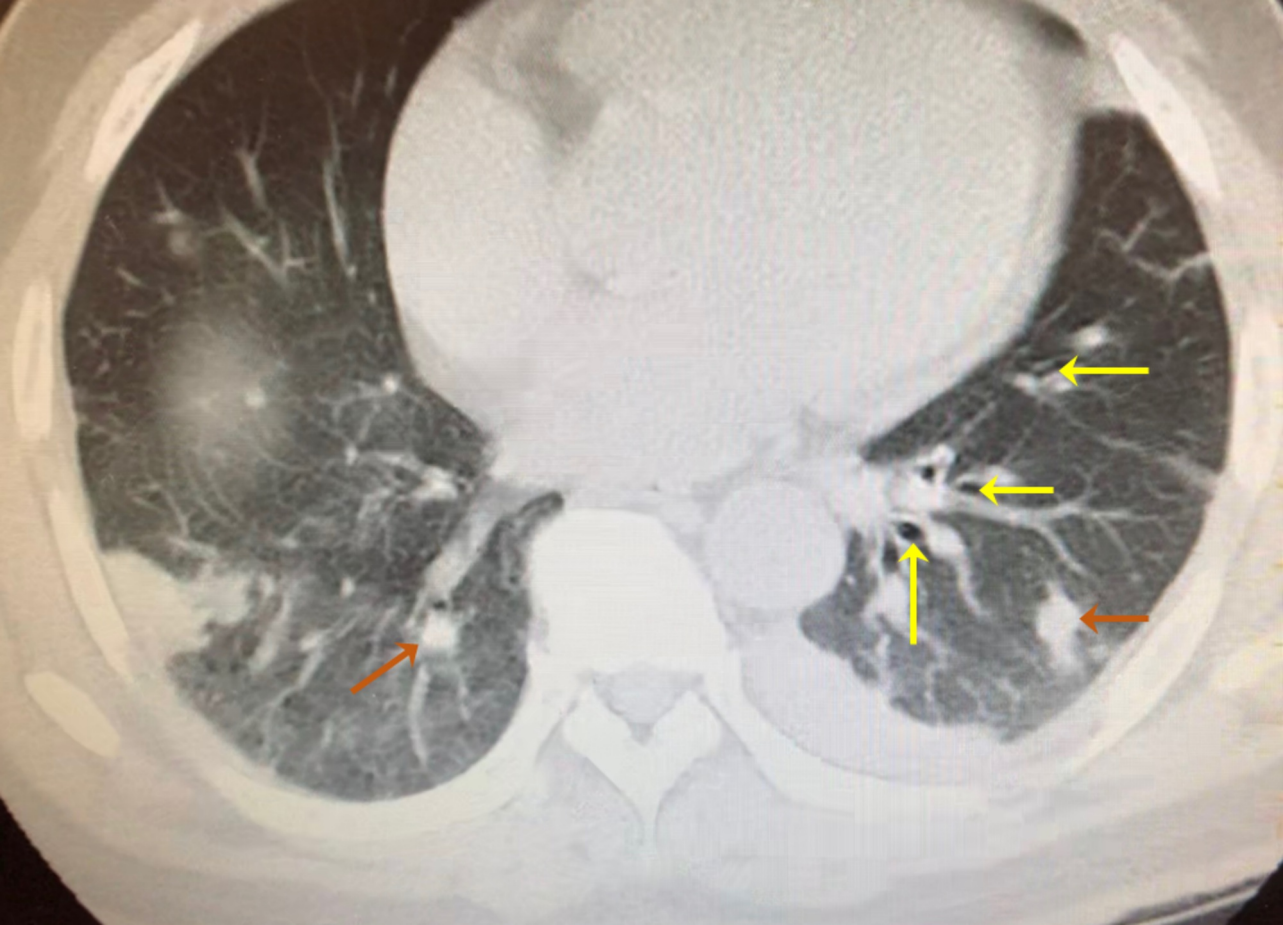
Chest CT of the patient at the time of tuberculosis diagnosis with air bronchograms (yellow arrows) and pulmonary nodules (orange arrows).

The patient is a 49-year-old Vietnamese male with a past medical history of latent TB and non-insulin-dependent diabetes mellitus type 2. He presented with nausea, vomiting, and progressively decreasing urine output for one week. The patient described his urine color dark brown like “Coca-Cola”. He had associated symptoms of generalized weakness and lower back pain. About two months before the current presentation, he was admitted to the hospital for pneumonia and diagnosed with latent TB. Chest CT at that time revealed multifocal pulmonary nodules or nodular consolidations widely distributed throughout each lung area. The consolidations were largest in the perihilar portion of the right lung and throughout the dependent portion of the left lung base that contained numerous internal air bronchograms (Figure 1).

CT-guided biopsy of a right pulmonary nodule was negative for malignancy or granuloma without any evidence of focal necrosis. Due to his country of origin in Southeast Asia, TB was suspected. Both sputum and bronchoalveolar lavage samples taken from broncoscopy were negative for acid-fast bacilli (AFB) stain or cultures. His purified protein derivative test revealed an induration of 31 mm, and the QuantiFERON®-TB Gold test was positive. He did not have any active TB symptoms of fevers, cough, weight loss, or night sweats, etc. He was then diagnosed with latent TB and discharged with supervised TB treatment with rifampin 300 mg weekly and isoniazid 900 mg weekly with a local health department.

**Figure 2:**
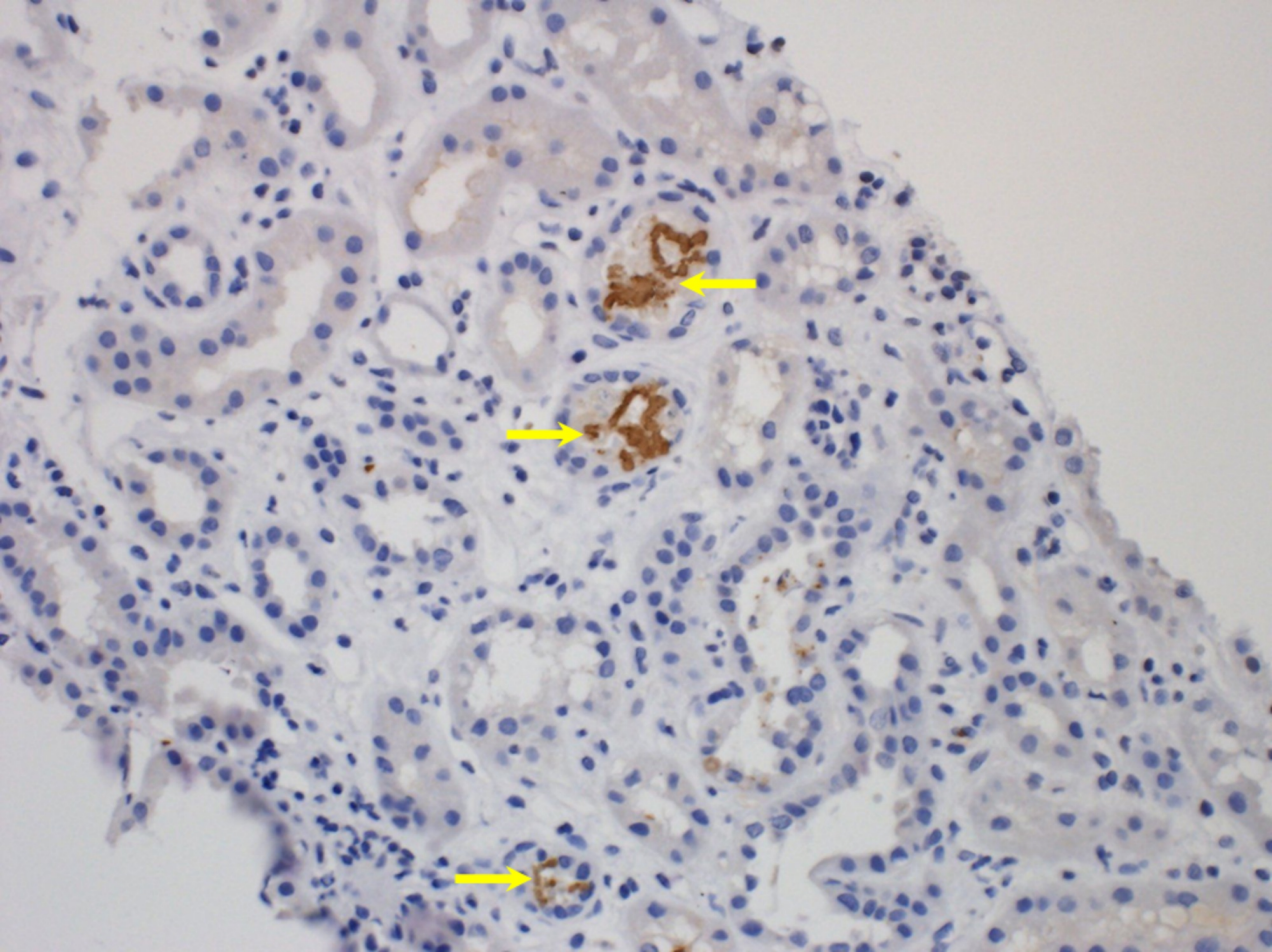
Immunoperoxidase stain showing hemoglobin containing brown casts inside three tubules (yellow arrows).

**Figure 3:**
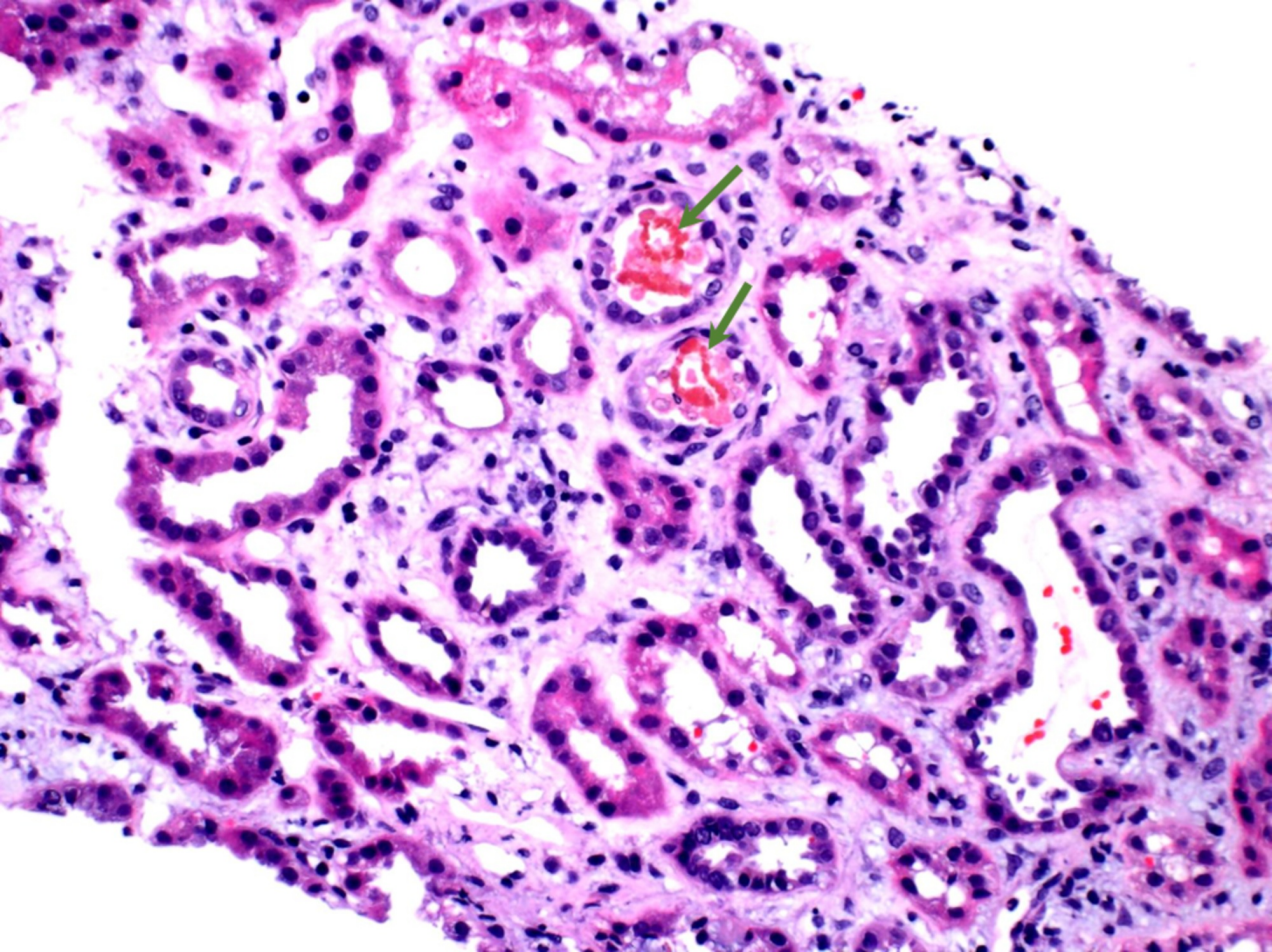
Hematoxylin and eosin stain of renal tubules containing pink red blood cell (RBC) casts (green arrows).

**Figure 4:**
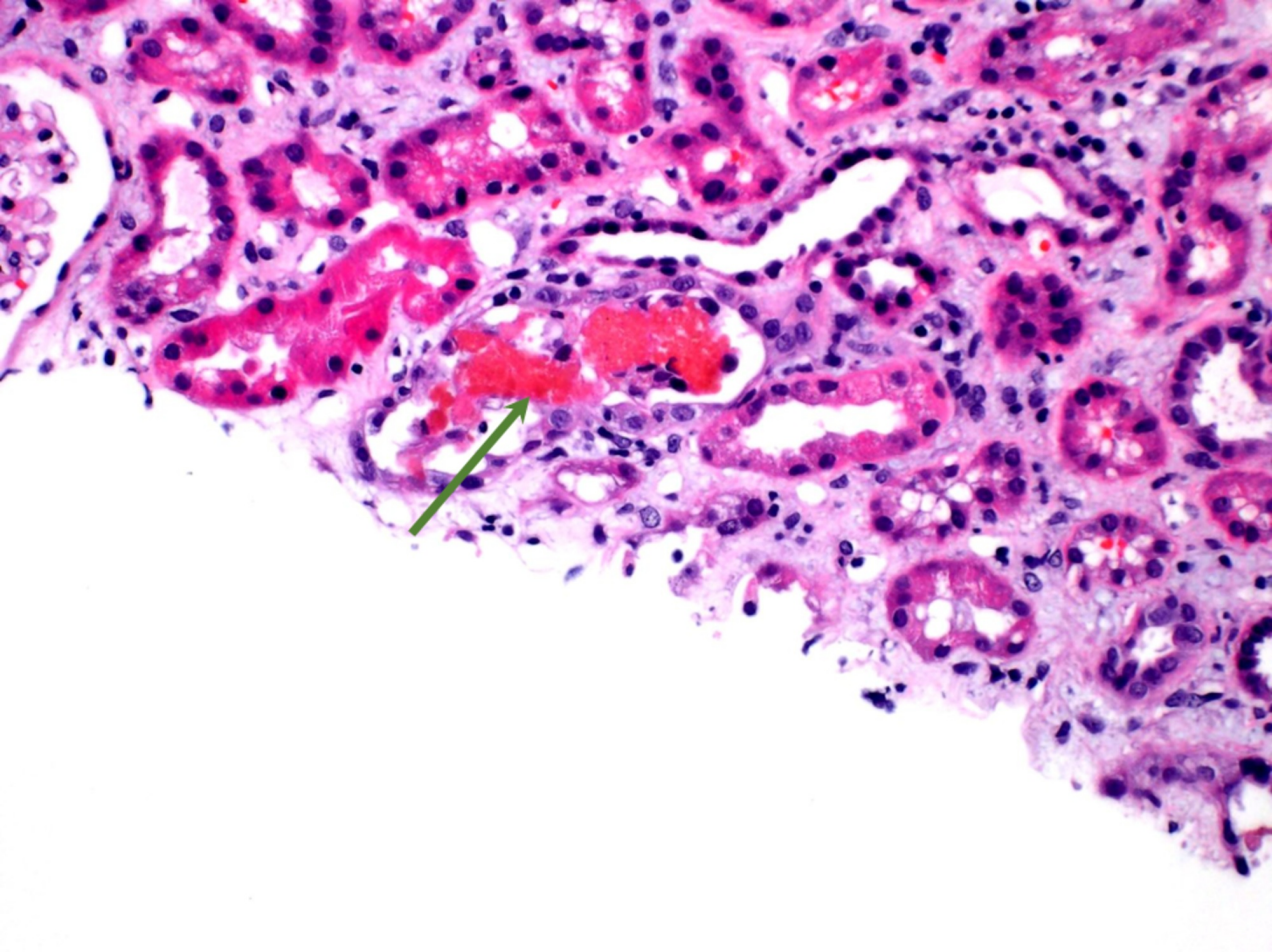
Red cell casts in tubules with damaged tubular epithelium (green arrow).

About two weeks before the current presentation, he missed taking rifampin and isoniazid due to the unavailability of a home health nurse. On the day of his current presentation, he had mild leukocytosis with white blood cell (WBC) count of 11.6 x 10^9^/L, hemoglobin 13.4 g/dL, hematocrit 26.4%, platelet count 132 x 10^9^ /L, blood urea nitrogen (BUN) 133 mmol/L, creatinine 16.1 mg/dL, glomerular filtration rate (GFR) 3 mL/min/1.73 m^2^ (compared to baseline creatinine 0.7 mg/dL, GFR 95 mL/min/1.73 m^2^ two months ago), normal transaminases (total bilirubin 0.5 µmol/L, aspartate aminotransferase [AST] 13 units/L, alanine aminotransferase [ALT] 12 units/L), sodium 132 mEq/L, potassium 4.9 mmol/L, chloride 97 mEq/L, bicarbonate 18 mEq/L, glucose 208 mg/dL, and a fractional excretion of sodium (FENa) less than 1%. His renal ultrasound was unremarkable with normal-sized bilateral kidneys and the absence of hydronephrosis. CT chest findings in present admission were mostly unchanged from CT chest done in the previous admission two months ago. He did not report any symptoms of active TB comprising of fevers, cough, weight loss, or night sweats. On the day of admission, his home rifampin 300 mg weekly dose was re-administered after a drug-free interval of two weeks. During the first two days of admission, he received aggressive fluid hydration to address hypovolemia from vomiting. He started to exhibit clinical symptoms of volume overload without significant improvement in his renal function. He continued to remain oliguric, and hemodialysis was commenced. Over first four days after rifampin resumption, his hemoglobin-hematocrit continued to decline from 13.4 g/dL-26.4% to 8.5 g/dL-24%, respectively. His total bilirubin increased to 2.4 µmol/L with a direct component of the bilirubin value of 0.5 µmol/L. On day 4, since admission, his urine analysis revealed a pH of 7.5, urine protein 1+, urine occult blood 3+, and trace red blood cells (RBCs). The urinalysis was suspicious for hemoglobin or myoglobin pigment-related kidney injury due to the marked presence of urine occult blood while lacking a significant number of RBCs. His serum creatinine kinase (CK) was mildly elevated at 300 units/L that ruled out myoglobin-induced acute renal failure (ARF), i.e., rhabdomyolysis. Coombs test was positive, and lactic acid dehydrogenase (LDH) was 450 units/L. Based on published reports of RARF and convincing laboratory data, it was concluded that his acute renal injury was worsened by intravascular hemolysis triggered by the re-introduction of rifampin. Due to limited resources, we were not able to check the anti-rifampin antibodies in his serum. On the sixth day post rifampin administration, despite receiving three hemodialysis sessions, his renal function did not improve, and a renal biopsy was performed. His renal biopsy revealed acute tubular injury with intratubular hemoglobin casts, concerning of intravascular hemolysis and hemoglobinuria (Figures 2-4).

Rifampin was discontinued. Subsequently, after five more hemodialysis sessions, his renal function exhibited steady improvement with a decrease in serum creatinine and an increase in GFR. When his urine output increased over 50 cc/hour with concomitant near normalization of creatinine and GFR, his hemodialysis was discontinued. He was discharged home, and rifampin was permanently removed from the discharge medication list. His latent TB treatment from the previous admission comprising of dual therapy with rifampin and isoniazid was substituted for monotherapy with isoniazid for a total of six months. In subsequent outpatient follow-ups of over 10 months to date, his renal function has remained within normal limits.

## Discussion

Various mechanisms of DIIHA have been proposed: “immune complex” (e.g., quinine and quinidine), “drug adsorption” (e.g., penicillin and cefotetan), true RBC autoantibodies causing AIHA (e.g., methyldopa), and “membrane modification” (e.g., cephalothin) [1]. The first immune complex mechanism is where drug-antidrug antibody complexes attach to the surface membrane of RBCs and cause lysis by complement activation. The second drug adsorption mechanism is where drug coats the surface of the cell membranes and drug-specific IgG antibodies attach to the drug, leading to extravascular hemolysis where macrophages destroy the RBCs that are coated with IgG antibodies. The third autoantibody mechanism is where drugs trigger the production of true RBC autoantibodies. The fourth mechanism of membrane modification is where the RBC membrane structure can become affected by the nonimmunological association of the drug to their membranes.

The IgM and IgG antibodies involved in DIIHA are of two main types: the first type is drug-dependent, i.e., will only react with RBCs in vitro in the presence of the drug [1]. The second type of antibody is drug-independent, i.e., will react with RBCs in vitro without the presence of the offending drug. Such antibodies appear to be as RBC autoantibodies rather than antibodies to the drug [1]. A case study reports immune hemolysis in three patients exhibiting the simultaneous development of both drug-independent autoantibody and drug-dependent antibody in response to rifampicin treatment [10].

The most common cause of acute kidney injury triggered by rifampin has tubular damage manifesting as AIN, ATN, and light-chain proteinuria [4,7,11,12]. One less common cause noted for AKI is diffuse proliferative crescentic glomerulonephritis [11]. ATN is mainly immune-mediated and is related to the formation of rifampin IgG and IgM antibodies in previous exposure to the drug [7]. The rifampin-dependent antibodies interact with I-antigen expressed on the surface of RBCs and form immune complexes leading to complement-mediated hemolysis of RBCs [7,9]. The I-antigen is also expressed in renal tubular cells. Hence, ATN could be triggered by two independent mechanisms, through anti-rifampin antibodies targeting the I-antigen on RBCs causing intravascular hemolysis, or anti-rifampin antibodies targeting I-antigen on renal tubular cells directly causing their destruction. Intravascular hemolysis indirectly causes ATN through the release of heme pigment that is nephrotoxic to renal tubules [7].

Rifampin-related IgG or IgM antibodies, which lead to acute RBC lysis, renal tubular cell damage, platelet lysis, or worse, even DIC leading to death, are commonly referred to as “immunoallergic” events [5]. These immunoallergic events are mainly observed with the intermittent or interrupted use of rifampin. The anti-rifampin antibodies can be detected in the serum of up to 30% of patients after three to four doses of monthly intermittent therapy with rifampin. Among all the immunoallergic reactions noted with sporadic rifampin use, 75% of reactions were observed after five or fewer doses. Infrequent dosing of rifampin on either intermittent or interrupted schedules can result in sensitization with a rapid increase in antibody titer after repeat drug exposure [5,6]. In contrast, the daily administration of rifampin is believed to confer immunologic tolerance against these reactions due to continuous clearance of anti-rifampin antibody complexes [5]. An observational study recording RARF events due to discontinuous rifampin use noted that the drug-free interval ranged from 10 days to six years [12]. One case reports RARF occurred after an 18-year interval since last rifampin use [4].

In a case series of 25 patients diagnosed with RARF over 10 years, RARF cases constituted 2.5% of all cases of ARF [12]. Another case study reports 60 cases of RARF over a period of eight years, and it was found there was one death (1.66%) among the 60 patients, compared to a 20% mortality rate of all ARF cases hospitalized in the same period (P < 0.05) [13]. Frequently reported clinical symptoms associated with RARF include nausea and vomiting (72%), fever (45%), chills (43%), abdominal pain (40%), diarrhea (26%), jaundice (19%), lumbar pain (17%), and anemia (96%) [14]. The severity of RARF can be quantified with the length of the anuric phase [13]. The duration of anuric phase had a positive correlation between the number of hemodialysis sessions, creatinine levels at 30 days, and the degree of hypergammaglobulinemia. Patients with hypergammaglobulinemia had a significantly longer anuric phase (15.5 ± 4.2 days vs. 10.4 ± 7.7 days, respectively, P = 0.05). The duration of the anuric phase, on average, was 11.4 days ± 7 days, and an average of 4.8 ± 4.6 hemodialysis sessions were required [13,14]. The mortality was very low, and renal function recovery was complete among 40% of patients in 30 days from the onset, and among 96.6% of patients in 90 days from the start [13]. In RARF cases, due to ATN alone, steroid therapy is not warranted [14].

## Conclusions

Acute hematologic dyscrasias with or without ARF associated with interrupted and intermittent use of rifampin can be potentially life-threatening. Before prescribing rifampin, a careful prior history of its dosing schedule is warranted. The reinstitution of rifampin after a prior interrupted interval should ideally be avoided or at least carefully monitored. If the patient has any clinical or laboratory evidence of acute hemolysis, thrombocytopenia, ARF, DIC, or any immunologic reaction to post-rifampin administration, the drug should be promptly discontinued and substituted with an alternative drug regimen.
